# Supplementary material for: Developmental evolution of the forebrain in cavefish, from natural variations in neuropeptides to behavior
Source: eLife. 2018 Feb 6;7:e32808. doi: 10.7554/eLife.32808 (PMC5800845; doi:10.7554/eLife.32808)
Supplement: Transparent reporting form [file elife-32808-transrepform.docx]

***eLife’s* transparent reporting form**

We encourage authors to provide detailed information *within their submission* to facilitate the interpretation and replication of experiments. If you have any questions, please contact us: [editorial@elifesciences.org](mailto:editorial@elifesciences.org).

**Sample-size estimation**

- You should state whether an appropriate sample size was computed when the study was being designed
- You should state the statistical method of sample size computation and any required assumptions
- If no explicit power analysis was used, you should describe how you decided what sample (replicate) size (number) to use

Please outline where this information can be found within the submission (e.g., page numbers or figure legends), or explain why this information doesn’t apply to your submission:

In the absence of existing pilot studies, no effect size was pre-specified and therefore no sample size was predetermined.

However, let’s take Hcrt neuron numbers at 24hpf as a “post-experimental” pilot study to estimate effect size of fish phenotype (CF vs SF), using the sample-size calculator made available by the University of California San Francisco (<http://www.sample-size.net/>). Power analysis assuming normal distribution, s.d. equal to 10%, type 1 error (α) equal to 5% and power (1-β) equal to 80% indicates a sample size of seven control and seven experimental subjects. In all our reported experiments, the sample sizes are equal or higher than seven.

**Replicates**

- You should report how often each experiment was performed
- You should include a definition of biological versus technical replication
- The data obtained should be provided and sufficient information should be provided to indicate the number of independent biological and/or technical replicates
- If you encountered any outliers, you should describe how these were handled
- Criteria for exclusion/inclusion of data should be clearly stated
- High-throughput sequence data should be uploaded before submission, with a private link for reviewers provided (these are available from both GEO and ArrayExpress)

Please outline where this information can be found within the submission (e.g., page numbers or figure legends), or explain why this information doesn’t apply to your submission:

This information can be found pages 28 of the manuscript.

**Statistical reporting**

- Statistical analysis methods should be described and justified
- Raw data should be presented in figures whenever informative to do so (typically when N per group is less than 10)
- For each experiment, you should identify the statistical tests used, exact values of N, definitions of center, methods of multiple test correction, and dispersion and precision measures (e.g., mean, median, SD, SEM, confidence intervals; and, for the major substantive results, a measure of effect size (e.g., Pearson's r, Cohen's d)
- Report exact p-values wherever possible alongside the summary statistics and 95% confidence intervals. These should be reported for all key questions and not only when the p-value is less than 0.05.

This information can be found pages 28 in the Methods section of the manuscript and directly on the figures (N, p-values). All figures show box plots, including the dispersion of the data.

Please outline where this information can be found within the submission (e.g., page numbers or figure legends), or explain why this information doesn’t apply to your submission:

(For large datasets, or papers with a very large number of statistical tests, you may upload a single table file with tests, Ns, etc., with reference to page numbers in the manuscript.)

**Additional data files (“source data”)**

- We encourage you to upload relevant additional data files, such as numerical data that are represented as a graph in a figure, or as a summary table
- Where provided, these should be in the most useful format, and they can be uploaded as “Source data” files linked to a main figure or table
- Include model definition files including the full list of parameters used
- Include code used for data analysis (e.g., R, MatLab)
- Avoid stating that data files are “available upon request”

Please indicate the figures or tables for which source data files have been provided:

For figures 2-3-4 (systematic neuron counts in cavefish and surface fish at several stages of development and for 9 neuropeptidergic cell types), the N values are not indicated on the figures that are already very dense.

The raw data (cell counts and replicates) are given in the Supplemental Table 1.
